# The influence of psychiatric screening in healthy populations selection: a new study and meta-analysis of functional 5-HTTLPR and rs25531 polymorphisms and anxiety-related personality traits

**DOI:** 10.1186/1471-244X-11-50

**Published:** 2011-03-31

**Authors:** Alessandra Minelli, Cristian Bonvicini, Catia Scassellati, Riccardo Sartori, Massimo Gennarelli

**Affiliations:** 1 Genetic Unit, I.R.C.C.S. "San Giovanni di Dio" - Fatebenefratelli, Brescia, Italy; 2 Department of Philosophy, Pedagogy, Psychology, University of Verona, Verona, Italy; 3 Department of Biomedical Sciences and Biotechnologies, Biology and Genetic Division, University School of Medicine, Brescia, Italy

**Keywords:** Neuroticism, Harm Avoidance, 5-HTTLPR, rs25531, meta-analysis

## Abstract

**Background:**

A genetic liability for anxiety-related personality traits in healthy subjects has been associated with the functional serotonin transporter promoter polymorphism (5-HTTLPR), although the data are somewhat conflicting. Moreover, only one study has investigated the functional significance of the 5-HTTLPR/rs25531 haplotypes in relation to anxiety traits in healthy subjects. We tested whether the 5-HTTLPR polymorphism and the 5-HTTLPR/rs25531 haplotypes are linked to Harm Avoidance (HA) using an association study (STUDY I) and a meta-analytic approach (STUDY II).

**Methods:**

STUDY I: A total of 287 unrelated Italian volunteers were screened for DSM-IV Axis I disorders and genotyped for the 5-HTTLPR and rs25531 (A/G) polymorphisms. Different functional haplotype combinations were also analyzed. STUDY II: A total of 44 studies were chosen for a meta-analysis of the putative association between 5-HTTLPR and anxiety-related personality traits.

**Results:**

STUDY I: In the whole sample of 287 volunteers, we found that the SS genotype and S'S' haplotypes were associated with higher scores on HA. However, because the screening assessed by Mini-International Neuropsychiatric Interview (M.I.N.I.) showed the presence of 55 volunteers affected by depression or anxiety disorders, we analyzed the two groups ("disordered" and "healthy") separately. The data obtained did indeed confirm that in the "healthy" group, the significant effects of the SS genotype and S'S' haplotypes were lost, but they remained in the "disordered" group. STUDY II: The results of the 5-HTTLPR meta-analysis with anxiety-related traits in the whole sample confirmed the association of the SS genotype with higher anxiety-related traits scores in Caucasoids; however, when we analyzed only those studies that used structured psychiatric screening, no association was found.

**Conclusions:**

This study demonstrates the relevance to perform analyses on personality traits only in DSM-IV axis I disorder-free subjects. Furthermore, we did not find an association between functional serotonin transporter gene polymorphisms and anxiety traits in healthy subjects screened through a structured psychiatric interview.

## Background

Personality traits can be defined as individual qualities or characteristics that influence cognitions, emotions, and behaviors and lead to adaptive or maladaptive responses. Human personality is a multidimensional structure that is affected by both environmental and genetic factors. According to the literature, individual variation of the heritable component is estimated to account for 30-40% of the variance in personality traits [1]. To date, the most frequently studied candidate gene for personality traits has been the functional polymorphism 5-HTTLPR in the promoter region of the SLC6A4 gene, which encodes the serotonin transporter. This polymorphism results in a short (S) and a long (L) variant [2,3].

Functional studies of the activity of the SLC6A4 promoter in transfected cell lines, postmortem human brains, and lymphoblasts have confirmed that the L allele is associated with higher levels of transcriptional activity and influences the rate of serotonin uptake more than the S variant [4]. According to recent findings, the S allele is associated with a less favorable response/resistance to pharmacological treatment [5-8] but also with central stress regulation [9-11].

Recently it has been critically discussed that the analysis of 5-HTTLPR is incomplete, because other polymorphisms have been found in the proximity of the Ins/Del locus, such as rs25531, rs25532, rs2020933, and a 17-bp variable tandem repeat in the second intron (STin2) [4,12,13]. In particular, rs25531, the polymorphism nearest 5-HTTLPR, results in an A to G substitution and has been shown to modulate the effect of 5-HTTLPR on transcriptional efficacy. Our recent work [8] reported evidence that the rs25531 polymorphism is located immediately outside of the 5-HTTLPR segment, resulting in the status of 5-HTTLPR and rs25531 as two independent polymorphisms. It has been reported that the G allele of rs25531 is in phase with the 5-HTTLPR long allele and mitigates transcriptional efficacy more than does the 5-HTTLPR short allele. Therefore, the modulation of 5-HTTLPR by rs25531 results in haplotypes with a high (L_A_) or low (L_G_, S_A _or S_G_) transcriptional efficacy [4,14].

The inventories mostly used in biological studies of personality are the NEO-Personality Inventory [15] and the Temperament and Character Inventory (TCI) [16]. Although NEO and TCI have relevant differences, they appear similar when evaluating anxiety traits such as Neuroticism (N) and Harm Avoidance (HA). Several studies have shown that N is highly related to HA [17,18], but there is evidence that N and HA may not be equivalent [19].

Since the first paper of Lesch [3] was published, a large number of studies have sought evidence of an association between the 5-HTTLPR polymorphism and anxiety-related personality traits. Despite these investigations, the strength and nature of any association is still uncertain. Controvertible results were obtained using both the TCI and NEO scales. In addition, five meta-analyses [20-24] provided conflicting results. In 2003, Munafò [20] reported an association between the 5-HTTLPR polymorphism and avoidance traits, but this effect was no longer being significant when data from studies reporting allele frequencies not in Hardy-Weinberg equilibrium (HWE) and unpublished data were excluded. Two successive meta-analyses [23,24] found an association between N and 5-HTTLPR, although no link with HA was observed. However, opposing data were reported in a different meta-analysis in 2005 [21]. Munafò et al. [21] concluded that the effect, if present, is small. More recently, the same authors [22] presented a more complete meta-analysis, which evidenced no association of 5-HTTLPR with HA and a significant association with N; however, the association was lost due to high between-study heterogeneity in analyses conducted using the random effects model.

It is important to emphasize that these contrasting results may be explained by the inclusion of studies that recruited participants from psychiatric populations. Both Schinka and Sen's meta-analyses [23,24] included data from healthy and patients whereas the different Munafò's meta-analyses [20-22] explicitly excluded studies that recruited from psychiatric populations and, when both psychiatric and control samples were recruited, data from healthy controls only were included. Indeed, the personality traits of pathological people could be confounding factors. It has long been known that depression and anxiety disorders are associated with higher scores for anxiety-related traits [25-28]. In addition, a higher frequency of the S allele was observed in depressed and anxious disorders patients [29]. Another relevant bias could be the inclusion of data from presumably healthy subjects without any psychological screening to exclude any DSM-IV axis I psychiatric disorders.

On the basis of these conflicting evidences we performed the following analyses: 1) STUDY I: an association study between the 5-HTTLPR and rs25531 and the relative estimated/phased haplotypes with anxiety personality traits measured using the self-rated TCI scale. The analyses were carried out in the whole sample of controls as well as in subjects without any DSM-IV axis I disorders screened by structured interviews; 2) STUDY II: meta-analyses of 5-HTTLPR and HA or N in controls and in screened samples.

**STUDY 1**: A new association study of 5-HTTLPR and 5-HTTLPR/rs25531 with HA

## Methods

### Participants

A total of 287 unrelated volunteers (age: 50.05 ± 15.94 years [mean ± SD]; range: 22 to 87 years; 117 males and 170 females) were recruited through a variety of sources, such as universities, newspaper advertisements, and elderly associations. The study protocol was approved by the Ethics Committee of Fatebenefratelli Hospital (San Giovanni di Dio, Brescia, Italy), and written informed consent was obtained from all the subjects. The subjects were screened for DSM-IV Axis I disorders through the Mini-International Neuropsychiatric Interview (M.I.N.I.) [30] and screened for any history of drug or alcohol abuse or dependence by expert psychologists. Personality traits were assessed by the Italian version of TCI, a 240-item true-false self-report questionnaire [31]. Subjects who scored less than 27/30 on the Mini-Mental State Examination (M.M.S.E.) [32] were excluded from the study to avoid biases in the completion of the TCI.

### Genotyping analyses of 5-HTTLPR and rs25531

Isolation of DNA, genotyping of 5-HTTLPR and rs25531, as well as the classification of estimated phased haplotypes (S_A_S_A_, S_G_S_G_, L_G_S_A_, and L_G_L_G _as S'S'; L_A _S_A _and L_A_L_G _as L'S'; and L_A_L_A _as L'L') were described in a study by Bonvicini [8]. We did not detect the L_G_L_G _or S_G_S_G _haplotypes in the genotyping analyses.

### Statistical analysis

The association between TCI scores and 5-HTTLPR or 5-HTTLPR/rs25531 was analyzed by analysis of variance, using the HA score as the dependent variable, genotypes and sex as independent variables, and age as a covariate (ANCOVA). The p-values have been corrected for multiple comparisons. All analyses were conducted using SPSS statistical software version 12.0 (SPSS Inc., Chicago, IL).

The association study showed a power > 80% by using the Quanto program version 1.2.4 with the following parameters: 5-HTTLPR S and S'S' minor allele frequencies (MAFs) of 15% and 21%, respectively, in a population of European origin; p ≤ 0.05; OR ≥ 1.6; log additive mode of inheritance; and population risk ≥ 10%.

## Results

In the whole sample of 287 volunteers, the minor S allele frequency of the 5-HTTLPR polymorphism was 0.39; the genotype frequencies and HA (mean score +/- SD) of LL, LS, and SS were 0.37 (43.42 +/- 17.14), 0.48 (42.55 +/- 17.96) and 0.15 (48.57 +/- 20.18), respectively (Table 1). The genotype distributions were in HWE (χ^2 ^= 0.05; p = 0.82). The results indicated a trend toward an association between 5-HTTLPR and anxiety-related scale for genotypes (p = 0.06), and a significant effect was found when we considered the L allele as dominant (p = 0.02). Concerning the analysis of the 5-HTTLPR/rs25531 the ANCOVA results showed an effect using a dominant L model (L'L' + L'S' vs. S'S' p = 0.05, Table 1).

**Table 1:** HA scores (Mean ± Standard Deviation) of all participants, including screened healthy subjects and people excluded for depression and anxiety disorders, stratified according to the 5-HTTLPR and the 5-HTTLPR/rs25531 estimated/phased haplotypes

	All subjects (287)			Healthy subjects (229)			**Disordered subjects **^**b **^**(55)**		
***Genotype 5-HTTLPR***	***N (freq. %)***	***HA (mean ± SD)***	***p (ANCOVA)***^**a**^	***N (freq. %)***	***HA (mean ± SD)***	***p (ANCOVA)***^**a**^	***N (freq. %)***	***HA (mean ± SD)***	***p (ANCOVA)***^**a**^
LL	107 (0.37)	43.42 ± 17.14	F = 2.84	93 (0.41)	41.63 ± 15.66	F = 0.34	13 (0.24)	57.14 ± 21.73	F = 1.85
LS	138 (0.48)	42.55 ± 17.96	p = 0.06	109 (0.48)	39.08 ± 16.72	p = 0.71	27 (0.49)	56.19 ± 16.88	p = 0.17
SS	42 (0.15)	48.57 ± 20.18		27 (0.11)	37.25 ± 12.11		15 (0.27)	68.95 ± 15.11	

***Carriers***									
Carriers L	245 (0.85)	42.93 ± 17.57	F = 5.73	202 (0.89)	40.25 ± 16.25	F = 0.18	40 (0.73)	56.50 ± 18.32	F = 3.86
vs SS			**p = 0.02**			p = 0.67			**p = 0.05**
Carriers S	180 (0.63)	43.95 ± 18.62	F = 0.37	136 (0.59)	38.72 ± 15.89	F = 0.70	42 (0.76)	60.75 ± 17.24	F = 0.60
vs LL			p = 0.54			p = 0.40			p = 0.44

***Phased Haplotype frequencies***									
L_A_L_A_	93 (0.32)	42.27 ± 16.60	F = 2.00	82 (0.36)	40.70 ± 14.97	F = 0.59	10 (0.18)	56.29 ± 23.41	F = 1.14
L_A_L_G_	14 (0.05)	51.02 ± 19.28	p = 0.09	11 (0.05)	48.57 ± 19.55	p = 0.67	3 (0.05)	60.00 ± 18.73	p = 0.35
L_A_S_A_	120 (0.42)	42.41 ± 17.58		94 (0.41)	38.85 ± 16.14		25 (0.46)	55.20 ± 17.16	
L_G_S_A_	18 (0.06)	43.49 ± 20.82		15 (0.07)	40.57 ± 20.58		2 (0.04)	68.57 ± 4.04	
S_A_S_A_	42 (0.15)	48.57 ± 20.18		27 (0.11)	37.25 ± 12.11		15 (0.27)	68.95 ± 15.11	

***Clustered Phased Haplotype frequencies***									
L'L'	93 (0.32)	42.27 ± 16.60	F = 2.12	82 (0.36)	40.70 ± 14.97	F = 0.04	10 (0.18)	56.29 ± 23.41	F = 2.50
L'S'	134 (0.47)	43.30 ± 17.88	p = 0.12	105 (0.46)	39.86 ± 16.69	p = 0.96	28 (0.51)	55.71 ± 17.03	p = 0.09
S'S'	60 (0.21)	47.05 ± 20.34		42 (0.18)	38.43 ± 15.50		17 (0.31)	68.91 ± 14.17	

***Carriers of Phased haplotypes***									
(L'L' + L'S')	227 (0.79)	47.05 ± 20.34	F = 3.92	187 (0.82)	38.43 ± 15.50	F = 0.08	38 (0.69)	68.91 ± 14.17	F = 5.15
vs (S'S')			**p = 0.05**			p = 0.77			**p = 0.03**
(L'S' + S'S')	194 (0.68)	44.46 ± 18.70	F = 1.47	147 (0.64)	39.45 ± 16.32	F = 0.04	45 (0.82)	60.70 ± 17.11	F = 0.81
vs L'L'			p = 0.23			p = 0.85			p = 0.37

The ANCOVA results for the genotypes, clustered genotype, and carriers are included.

^a ^HA score as the dependent variable, genotype or clustered genotype and sex as independent variables, and age as a covariate.

^b ^Disordered group consisted of subjects with depression and anxiety disorders.

Based on the assessment performed using M.I.N.I., the sample consisted of 229 (80%) subjects without lifetime DSM-IV Axis I disorders (the "healthy" group) and 58 subjects (20%) with these disorders (the "disordered" group). In the "disordered" group, 38 subjects had MDD, 2 had Panic Disorder, 22 had Generalized Anxiety Disorder, 6 had Dysthymia, 1 had Bipolar Disorder, 1 experienced alcohol abuse and 1 experienced substance abuse (the total number exceeds the number of subjects due to the presence of comorbidity). Because the literature has largely shown that people affected by unipolar major depression and anxiety disorders present homogeneous patterns of personality traits compared to other subjects [25-28,33-36], the 55 participants with depression and/or anxiety lifetime diagnosis were regrouped. The three subjects affected by Bipolar Disorder, experienced alcohol and substance abuse were consequently excluded.

Thus, to evaluate whether the results from the participants excluded by M.I.N.I. had influenced the previous analyses, we performed ANCOVA using the HA score as the dependent variable, groups ("healthy" N = 229, "disordered" N = 55), genotypes, and sex as independent variables, and age as a covariate for both 5-HTTLPR and estimated/phased haplotypes. The results indicated that, the disordered group showed significantly higher HA scores than healthy subjects (F = 46.72, p < 0.0001). No association was found between 5-HTTLPR polymorphism and anxiety traits (F = 1.34, p = 0.26), whereas a significant interaction was observed between the 5-HTTLPR genotype and groups (F = 4.52, p = 0.03). The same pattern was obtained when the SS genotype was compared to allele L carriers (F = 4.41, p = 0.04). Concerning the 5-HTTLPR/rs25531, a significant interaction was detected with the dominant L model (L'L' + L'S' vs. S'S'; p = 0.02). In all analyses, no significant gender effect or interaction was obtained.

In the sample of subjects with no DSM-IV axis I disorders (healthy group), we performed an ANCOVA analysis to test the possible association between polymorphisms and HA. There was no significant association between HA and either the 5-HTTLPR or the 5-HTTLPR/rs25531 haplotypes (Table 1). Despite its small size, the disordered group revealed an association between HA and SS or S'S' homozygosity (p = 0.05 and p = 0.03, respectively).

**STUDY 2**: Meta-analyses of 5-HTTLPR with anxiety traits

## Methods

### Literature search

To identify eligible studies for the meta-analysis, we performed a search through PubMed (at the National Library of Medicine) for all the available studies of the association between the serotonin transporter and anxiety personality traits conducted in healthy adults, using the following search terms: serotonin transporter polymorphism, serotonin transporter gene, 5-HTTLPR, Neuroticism, Harm Avoidance, anxiety, and personality. Once articles had been collected, bibliographies were manually searched for additional eligible studies.

### Inclusion criteria

All association studies that have measured anxiety traits using any version of NEO (NEO-PI, NEO-PI-R, or NEO-FFI) or the TCI (or TPQ) in male and/or female participants of any ethnic origin were included. Only data from controls were included from studies in which psychiatric patients and control data were compared. Data that appeared in more than one published study were included only once in the analyses. Papers not written in English [37] were excluded.

### Data extraction

We recorded the number of participants, the mean of N and/or HA trait scores, and the standard deviation for each of the three genotype groups (LL, LS, SS) in each study included in our analysis. Furthermore, we extracted data regarding the male/female ratio, the mean age, the ethnic compositions of the sample, and the structured clinical interview used for screening. Genotype frequencies were used to calculate the HWE (program http://www.genemapping.cn). In cases where all or part of this information was not available in the publication, the authors were contacted by email.

### Statistical analyses

The Review Manager was used to analyze data (RevMan Version 5.0.16; Copenhagen, The Nordic Cochrane Centre, The Cochrane Collaboration, 2008).

Firstly, data were analyzed with the fixed effects model in order to combine individual study effect sizes (Cohen's *d*s) using inverse variance methods to generate a summary *d *and 95% confidence interval (CI). We analyzed a possible association by both comparing LL genotype versus carriers of the S allele and SS genotype versus carriers of the L allele. The significance of the pooled effect sizes was determined by the *Z*-test and the between-study heterogeneity was assessed using a χ^2 ^test of goodness of fit and I^2 ^statistic [38]. The significant *p *value was set at 0.05. In a fixed effects model, the fundamental assumption is that a single true effect size underlies all study results and that observed estimates vary only as a function of chance. The error term in a fixed effects model represents only within-study variation, and between-study variation is ignored. Where the results showed a significant effect in the presence of significant between-study heterogeneity, a random effects model was utilized, with *d*s pooled using the DerSimonian and Laird methods [39]. In contrast, a random effects model assumes that each study estimates different, yet related, true effects and that the distribution of the various effects is normally distributed around a mean effect size value. This model takes both within- and between-study variation into account. When there is little heterogeneity, both models yield essentially identical results. When heterogeneity is extensive, however, the analyses will yield different estimates of the mean effect size, and the confidence intervals around the estimates will be different sizes. When there is heterogeneity across studies, the random effects model yields wider confidence intervals than the fixed effects model and is thus usually more conservative.

## Results

A total of 50 studies [3,22,40-88] met our inclusion criteria; their features are shown in Table 2. Six studies were excluded from our analyses for significant deviation from HWE (p ≤ 0.05) [46,52,60,65,74,81], and one was excluded for excessive ethnic heterogeneity [56]. Furthermore, nine other studies [50,62,63,67,68,71,76,78,86] were not included because the data regarding anxiety traits for each genotype and/or the data to test HWE were insufficient, and we were unable to obtain this kind of information from the authors.

**Table 2:** Characteristics of association studies eligible for inclusion

Study	Year	**Inventory**^**a**^	N	% Male	Mean Age	Ethnicity	HW equilibrium	HW χ2	HW p	Exclusion
Lesch	1996	NEO	505	92	37.6	94% Caucasian	YES	0.01	0.93	
Ebstein	1997	TCI	121	55	29.7	74% Caucasian	YES	1.14	0.29	
Nakamura	1997	Both	186	0	19.6	Asian	YES	1.15	0.28	
Mazzanti	1998	TCI	215	85	35.5	Caucasian	YES	0.01	0.98	
Ricketts	1998	TCI	37	nd	nd	Caucasian	YES	2.10	0.15	
Flory	1999	NEO	225	50	45.7	84% Caucasian	YES	0.16	0.69	
Hamer	1999	TCI	634	43	31.3	79% Caucasian	NO	3.85	0.04	Excluded^b^
Katsuragi	1999	TCI	101	61	25.0	Asian	YES	0.02	0.88	
Kumakiri	1999	Both	144	42	24.4	Asian	YES	1.28	0.26	
Benjamin	2000	TCI	455	40	nd	Caucasian	N/A			Excluded^d^
Comings	2000	TCI	81	100	32.9	Caucasian	YES	0.10	0.75	
Du	2000	NEO	186	41	36.3	Caucasian	YES	0.77	0.38	
Greenberg	2000	NEO	397	16	28.6	Caucasian	NO	4.75	0.03	Excluded^b^
Herbst	2000	TCI	425	51	43.8	67% Caucasian	YES	0.79	0.38	
Hu	2000	NEO	759	62	29.2	81% Caucasian	YES	1.57	0.21	
Osher	2000	Both	148	34	30.7	Caucasian	YES	0.11	0.75	
Schmidt	2000	NEO	72	48	27.0	54% Caucasian	YES	0.06	0.80	Excluded^c^
Samochowiec	2001	TCI	126	30	23.8	Caucasian	YES	1.26	0.26	
Cohen	2002	TCI	559	0	nd	Caucasian	NO	9.51	0.01	Excluded^b^
Tsai	2002	TCI	192	49	29.3	Asian	YES	2.30	0.13	
Brummett	2003	NEO	99	32	70.3	87% Caucasian	YES	0.70	0.40	
Umekage	2003	NEO	244	8	37.7	Asian	YES	2.08	0.15	
Ham	2004	TCI	146	32	31.9	Asian	YES	0.01	0.98	
Jacob	2004	Both	281	25	22.4	Caucasian	YES	0.59	0.44	
Lang	2004	NEO	228	50	38.6	Caucasian	YES	1.14	0.29	
Park	2004	TCI	100	0	48.3	Asian	YES	2.41	0.12	Excluded^d^
Samochowiec	2004	Both	100	47	41.0	Caucasian	YES	0.04	0.85	
Szekely	2004	TCI	151	43	22.2	Caucasian	YES	0.59	0.44	
Thierry	2004	TCI	76	0	32.8	Caucasian	YES	0.01	0.96	Excluded^d^
Sen	2004	NEO	415	33	43.8	Caucasian	NO	3.76	0.05	Excluded^b^
Bachner-Melman	2005	TCI	872	nd	21.4	N/A	N/A			Excluded^c,d^
Hariri	2005	TCI	92	49	30.5	Caucasian	N/A			Excluded^d^
Kim	2005	TCI	211	51	26.5	Asian	YES	0.06	0.81	Excluded^d^
Kremer	2005	TCI	730	nd	nd	N/A	N/A			Excluded^c,d^
Dragan	2006	NEO	196	0	21.7	Caucasian	YES	2.07	0.15	
Lazagorta	2006	TCI	57	nd	45	Other	YES	3.70	0.05	Excluded^b,c^
Monteleone	2006	TCI	94	0	nd	Caucasian	YES	2.37	0.12	
Serretti	2006	TCI	132	nd	nd	Caucasian	YES	0.02	0.90	
Vorfelde	2006	Both	195	50	nd	Caucasian	YES	0.48	0.49	
Hunnerkopf	2007	NEO	272	25	21.9	Caucasian	N/A			Excluded^d^
Joo	2007	TCI	158	44	23.8	Asian	YES	0.13	0.72	
Nilsson	2007	TCI	196	60	17	Caucasian	YES	1.07	0.30	
Schmitz	2007	Both	410	36	24	Caucasian	YES	0.07	0.78	
Stein	2008	NEO	247	31	18.8	61% Caucasian	NO	3.88	0.05	Excluded^b^
Lee	2008	TCI	75	100	16.1	Asian	YES	2.97	0.08	Excluded^d^
Kazantseva	2008	TCI	301	20	19.8	Caucasian	YES	1.24	0.26	
Suzuki	2008	TCI	575	51	28.7	Asian	YES	0.14	0.71	
Munafò	2009	TCI	3872	44	42	Caucasian	YES	0.26	0.61	
Gonda	2009	TCI	169	0	nd	Caucasian	YES	0.20	0.65	
Terracciano^e^	2009	NEO	3972	43	42.5	Caucasian	YES	1.33	0.25	
Terracciano^f^	2009	NEO	1182	52	57.3	71% Caucasian	YES	0.87	0.35	
Saiz	2010	TCI	404	50	40.5	Caucasian	YES	0.76	0.38	
Present Study		TCI	229	45	49.2	Caucasian	YES	0.33	0.56	

HW = Hardy-Weinberg; HW χ2 = Hardy-Weinberg chi square; HW p = Hardy-Weinberg *p *value; nd = not determined; N/A = not applicable.

^a ^The term NEO referred to all versions (i.e. NEO-PI, NEO-PI-R, NEO-FFI); the term TCI referred to all versions (TPQ).

^b ^Excluded because genotype frequencies showed deviation from Hardy-Weinberg equilibrium.

^c ^Excluded due to the ethnic heterogeneity or lack of data about ethnic origin.

^d ^Excluded because of unavailable data.

^e ^Data referred to SardiNIA sample.

^f ^Data referred to BLSA (Baltimore Longitudinal Study of Aging) sample.

Therefore, the meta-analysis used the results of 35 studies, including 7 [41,49,55,59,69,84,85] that reported data for both inventories, 1 [83] that generated data on NEO on 2 different independent samples, and the data of present work; in total, 44 samples were available for analysis.

**Figure 1:**
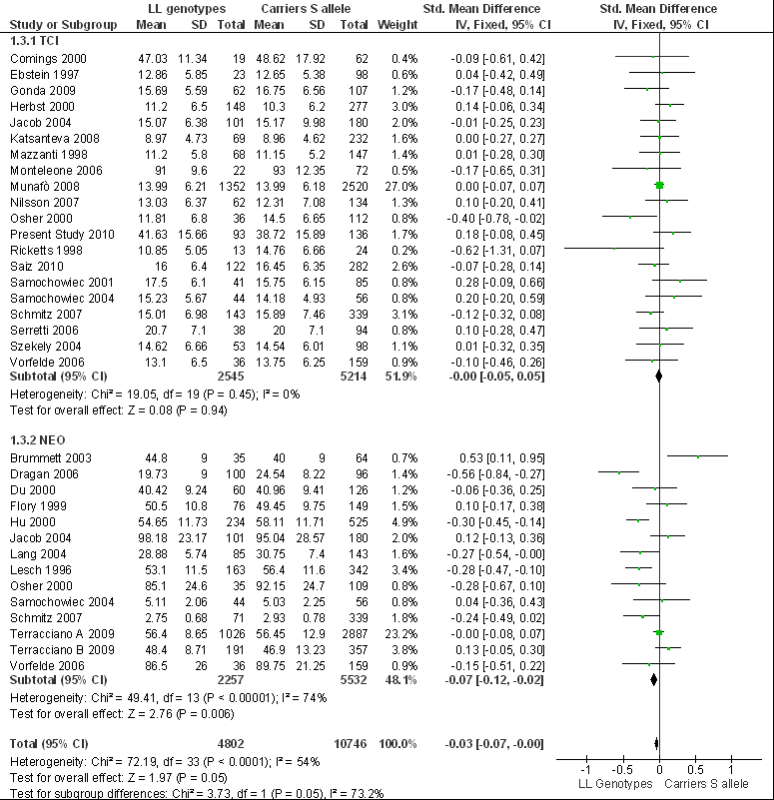
**Meta-analysis of 5-HTTLPR LL versus carriers S allele**. Meta-analysis of association studies of serotonin transporter gene and anxiety-related personality traits measured by NEO and TCI in Caucasoid population. It was used fixed effects model testing the comparison between LL genotype versus carriers S allele. Bars represent individual study 95% CI, with a central block proportional to study effect size, while summary diamond bar represents the pooled effect size estimate and 95% CI.

Because of ethnic differences in the 5-HTTLPR genotype distribution, the studies on Asian and Caucasoid populations were independently analyzed. When we conducted a comparison analysis between the LL genotype and S allele carriers in the Caucasoid population (Figure 1), no association was observed between 5-HTTLPR and HA (p = 0.94), and no evidence of between-study heterogeneity was apparent. A significant association with N (p < 0.01), indicating a higher anxiety trait score, and evidence of highly significant between-study heterogeneity (p < 0.0001, I^2 ^= 74%) were found in the S allele carriers group. When the analysis was run again using the random effects method, the significant effect just described was no longer significant. No evidence for an association between the 5-HTTLPR genotype and N (p = 0.09) as well as no overall effect (p = 0.11) was shown.

**Figure 2:**
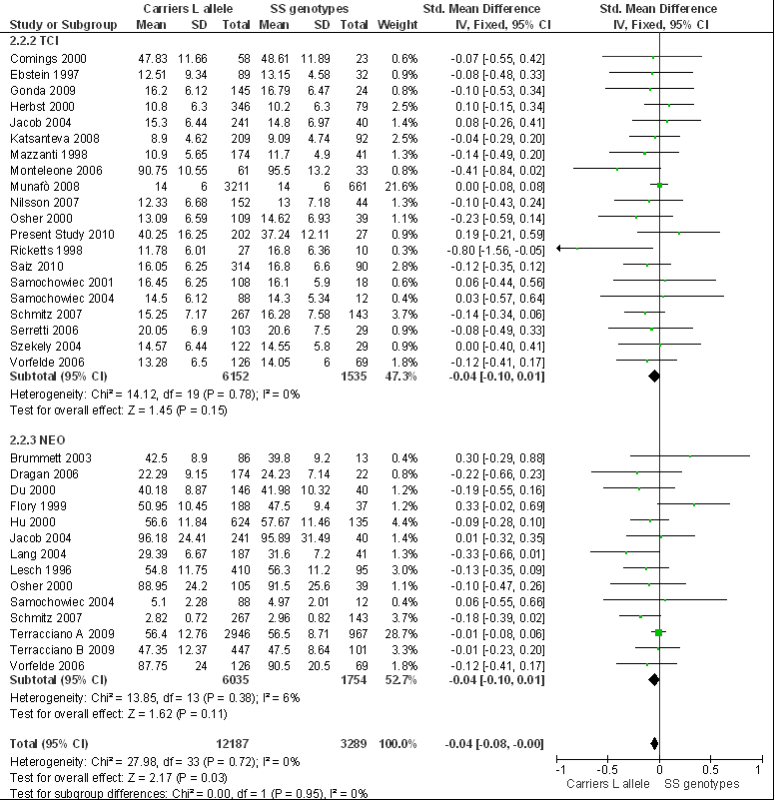
**Meta-analysis of 5-HTTLPR SS versus carriers L allele**. Meta-analysis of association studies of serotonin transporter gene and anxiety-related personality traits measured by NEO and TCI in Caucasoid population. It was used fixed effects model testing the comparison between carriers L allele versus SS genotype. Bars represent individual study 95% CI, with a central block proportional to study effect size, while summary diamond bar represents the pooled effect size estimate and 95% CI.

When we tested the L allele carriers versus the SS genotype in the same ethnic population (Figure 2), no association was found between 5-HTTLPR and HA or N, and there was no evidence of between-study heterogeneity. Instead, a significant overall effect was obtained (p = 0.03), and the two subgroups did not show significant differences (χ^2 ^[1] = 0.01, p = 0.95, I^2 ^= 0%).

In the Asian population, no association was observed between the LL genotype and carriers of the S allele, using either TCI (d = -0.01, 95% CI = -0.24, 0.22, Z = 0.10, p = 0.92) or NEO (d = -0.15, 95% CI = -0.54, 0.24, Z = 0.75, p = 0.46). After clustering of the L allele carriers versus the SS genotype, there was no significant evidence of an association between 5-HTTLPR and either HA or N (d = -0.06, 95% CI = -0.16, 0.04, Z = 1.13, p = 0.26; and d = -0.12, 95% CI = -0.29, 0.05, Z = 1.38, p = 0.17; respectively). We did not find between-study heterogeneity in any groups.

Because of the bias inherent in a mix of healthy subjects with depressed or anxious people, we carried out a meta-analysis including only the studies with structured psychiatric interview screening [42,43,59,66,73,75,88]. No significant result was found when we considered an L dominant model (TCI: d = 0.00, 95% CI -0.12, 0.12, Z = 0.01, p = 1.00; NEO: d = -0.02, 95% CI -0.22, 0.18, Z = 0.19, p = 0.85; Overall effect p = 0.92) or a recessive model (TCI: d = -0.10, 95% CI = -0.25, 0.04, Z = 1.40, p = 0.16; NEO: d = -0.12, 95% CI = -0.39, 0.14, Z = 0.93, p = 0.35; Overall effect p = 0.09).

## Discussion

The present study demonstrates the relevance of employing more stringent inclusion/exclusion criteria in association studies on healthy subjects. Our results show the influence of mistakes in the selection of subjects, underscoring the importance of the use of a structured psychiatric interview when people are enrolled as control subjects for this type of study. When we performed analyses on the whole sample of 287 volunteers, effects on the susceptibility to HA were found for both the SS genotype and the S'S' haplotypes. However, because the screening performed by M.I.N.I. revealed the presence of depression or anxiety disorders in 55 volunteers (the "disordered" group), we verified the possible influence of the variable "groups" ("healthy" and "disordered") on the genotypes. The results evidenced a significant interaction between genotypes and groups (5-HTTLPR; p = 0.03 and 5-HTTLPR/rs25531 L'L' + L'S' vs. S'S'; p = 0.02); thus, we conducted the analyses separately for these groups. The data confirmed that in the "healthy" group, the effects of the SS genotype and the S'S' haplotypes were lost, but they remained in the "disordered" group. In addition, we conducted a meta-analysis involving approximately 18,000 controls of Caucasoid and Asian descent and considering anxiety traits measured by TPQ/TCI or NEO. Similarly, an association was observed between S allele in homozygosity and higher scores for anxiety-related traits, but when we analyzed only the studies that used structured psychiatric screening, no association was found.

Moreover, another important finding from both STUDY I and STUDY II is the absence of a role of the serotonin transporter gene in anxiety personality traits in healthy subjects.

To date, five meta-analyses have been conducted on the involvement of the functional 5-HTTLPR polymorphism with anxiety personality traits [20-24]. Schinka's and Sen's meta-analyses [23,24] found a strong association of 5-HTTLPR with N and no link to HA, whereas Munafò [20-22] reported contrasting data; in particular no strong effect was detected [20,22] and, when present, it was small [21]. As discussed in the 2005 study by Munafò [21], the association found in Schinka and Sen's meta-analyses [23,24] may have been biased by the inclusion of studies that recruited participants from psychiatric populations. However, there is another bias that the Munafò meta-analyses [20-22] did not take in consideration: the presence of studies in which structured psychiatric screening was not performed, producing a lack of information about the patients' lifetime history of psychiatric disorders. On this basis, we tried to verify whether the absence of a psychiatric screening interview might represent an important confounding variable in studies regarding the biological basis of personality traits in healthy populations. Indeed, STUDY I indicated an association between the SS genotype and S'S' haplotypes in 5-HTTLPR/rs25531 and anxiety traits in the whole sample of volunteers, but these effects were probably found due to the presence of subjects with depression and anxiety disorders. In fact, as reported in Table 1, these subjects have higher scores for HA and a higher frequency of homozygous SS or S'S'. More importantly, the results of STUDY II lead to the same direction.

It is well known that anxiety traits are strongly linked to depression and anxiety disorders [25-28,33-36,89] and indeed, the premorbid depressive personality represents an emotional vulnerability that increases the likelihood of developing these disorders during stressful life events. Furthermore, the literature supports the hypothesis that 5-HTTLPR S allele could be a risk factor for major depression/anxiety spectrum disorders [29]. Taking together the two issues, our findings seem to be contradictory. However, recently it has been proposed the hypothesis about a role of the SLC6A4 gene not directly in the MDD susceptibility but rather in the some features of the pathology such as the response/resistance to antidepressant treatment [5-8], or the interaction with the stressful life events, given the robust correlation between these events and risk of developing depressive symptoms [9-11,90].

In our recent paper [8], we have supported the evidence about the modulation of 5-HTTLPR by rs25531 showing that L_G _haplotype has lower transcriptional efficacy as well as the S allele. Therefore, in STUDY I we conducted association analyses for 5-HTTLPR/rs25531 to investigate the influence of rs25531. The results showed the association with HA in the "disordered group". In light of these data, we speculate that the genotyping of both the functional polymorphisms (5-HTTLPR and rs25531) and the haplotypes analysis should be taken into account in relation to anxiety-related personality traits.

Finally, in STUDY II, because the S allele is much more prevalent in Asians than in Caucasians [41,49,58], suggesting that ethnic differences may be a confounding factor in association studies of the 5-HTTLPR genotype, we conducted separate analyses for both populations to avoid biased conclusions. No significant association was found between 5-HTTLPR and either N or HA.

## Conclusions

This study supports the following conclusions: 1. A lack of structured psychiatric screening of subjects may produce an important bias in genetic association studies on personality traits using controls. The symptomatology of depressive and anxiety disorders might interfere with anxiety-related traits in possible associations with the serotonin transporter and the higher frequency of the S allele observed in depressed and anxiety disorder patients; 2. The SLC6A4 gene is not involved in anxiety-related traits measured by TCI and NEO in psychiatrically healthy subjects.

## Conflict of interests

The authors declare that they have no competing interests.

## Authors' contributions

AM conceived of the study, participated in its design and the coordination and acquisition of data, performed the statistical analyses, and co-wrote the manuscript; CB participated in the design of the study, performed the statistical analyses and carried out all genetic analyses; CS participated in the design and coordination of the study and co-wrote the manuscript; RS performed the statistical analyses and helped draft the manuscript; MG conceived of the study, participated in its design and coordination, and helped draft the manuscript and critically reviewed it for intellectual content. All the authors read and approved the final manuscript.

## Pre-publication history

The pre-publication history for this paper can be accessed here:

http://www.biomedcentral.com/1471-244X/11/50/prepub
